# Effect of Doum (*Hyphaene thebaica*) mesocarp and endosperm extracts on Triton X-100 induced hyperlipidemic rat: mitigative cardiovascular and renal dysfunction risks

**DOI:** 10.3389/fnut.2026.1748885

**Published:** 2026-03-11

**Authors:** Munirah S. Almezail, Raghad M. Alhomaid

**Affiliations:** Department of Food Science and Human Nutrition, College of Agriculture and Food, Qassim University, Buraydah, Saudi Arabia

**Keywords:** antioxidant activity, cardiovascular diseases, endosperm, hyperlipidemia, *Hyphaene thebaica*, mesocarp, renal dysfunction

## Abstract

**Introduction:**

Hyperlipidemia increases the risk of cardiovascular (CVD) progression and renal dysfunction.

**Objectives:**

This study aimed to identify, for the first time, the protective effects of both the *Hyphaene thebaica* (Doum) edible mesocarp (DM) and inedible endosperm (DE) on hyperlipidemia and its associated cardiovascular and renal risks.

**Methods:**

Wistar rats were divided into seven groups: a negative control, a Triton X-100 (TrX-100)-induced hyperlipidemic model group, a group treated with atorvastatin, and groups receiving DM or DE at doses of 500 or 1,000 mg/kg alongside TrX-100 induction.

**Results:**

The TrX-100 model group exhibited significant hyperlipidemia, characterized by elevated triglycerides, total cholesterol, low-density lipoprotein, and total lipids. This was accompanied by increased CVD risk indicators (coronary artery index, cardiac index, atherogenic index, and angiotensin-converting enzyme), reduced kidney tissue antioxidants, increased malondialdehyde, and elevated markers of renal dysfunction (creatinine, urea, and uric acid). Administration of a high dose of DM (1,000 mg/kg) exerted significant hypolipidemic effects, mitigated CVD risk factors, protected renal function, and rebalanced kidney tissue antioxidant activities and malondialdehyde levels. Meanwhile, a dose of 500 mg/kg DM showed a mild effect on the lipid profile and factors mitigating CVD risk, kidney dysfunction, and kidney tissue antioxidants. Interestingly, a low dose of DE (500 mg/kg) had a more pronounced hypolipidemic impact, significantly mitigating CVD risk and enhancing kidney tissue antioxidant activities, compared to a high dose. Histopathological observation of kidney specimens confirmed the findings of kidney function.

**Conclusively:**

DM at a high dose achieved the best hypolipidemia effect, the highest protection against CVD risk, and improved renal function. The performance of DM was dose-dependent. A Low dose of DE had a more pronounced effect than a high dose, making it a point for future research on DE safety and reliability.

## 1 Introduction

Hyperlipidemia is characterized by a decrease in high-density lipoprotein cholesterol (HDL-C) and an increase in blood lipid profiles, specifically low-density lipoprotein cholesterol (LDL-C) and triglycerides (TGs) (1). Hyperlipidemia is a major public health issue that has been increasing globally due to poor eating habits, lifestyles, and genetic predispositions (2). A contributing factor for the development of atherosclerotic plaques and subsequent cardiovascular diseases (CVDs) (3) and renal dysfunction (4) is hyperlipidemia. Both CVDs and renal dysfunction contribute substantially to global morbidity and mortality, which represent 30% of global deaths (5). Rising LDL-C is the best indicator of atherosclerosis risk, as it contributes to the development of arterial atherosclerotic plaques, ultimately leading to CVDs such as myocardial infarction (6).

The relationship between hyperlipidemia and renal dysfunction is well-documented in some research (7). Hyperlipidemia is a risk factor for the progression of chronic kidney disease (CKD) (8). Hyperlipidemia has been shown to induce glomerular damage, characterized by glomerulosclerosis and tubulointerstitial fibrosis, which accelerates the progression of renal dysfunction (4). However, conflicting results have been reported in different studies. Clinical trials and observational cohorts in established CKD patients have failed to establish a direct, independent relationship between hyperlipidemia and the incidence of renal dysfunction (9). This discrepancy underscores the need for further studies on the role and metabolism of blood lipids in renal function.

The pharmacotherapeutic interventions commonly used in hyperlipidemia cases are statins (atorvastatin and lovastatin), which are known as HMG-CoA reductase inhibitors. However, despite their benefit in decreasing LDL-C levels and minimizing CVD risk, statins are not without side effects (1). Statins may cause some adverse symptoms, which include muscle pain with cramps and renal injury or death (3, 10). Therefore, using natural products with lipid-lowering properties with fewer side effects is becoming a growing trend.

Triton X-100 (TrX-100) is a non-ionic surfactant, well-known for its function in model-induced hyperlipidemia. It promotes intestinal lipid absorption by emulsification and accelerates cholesterol synthesis in the liver (11). It has been successfully used to induce hyperlipidemia in rats (12), and it was chosen in the present study as the hyperlipidemic model due to its convenience, reproducibility, and availability.

*Hyphaene thebaica* (Doum) is a dioecious palm belonging to the Arecaceae family (Palmae) native to Egypt, Sudan, and the Arabian Peninsula (13). Doum’s strong phytochemical composition has made it a promising candidate with diverse therapeutic properties (13, 14). Doum consists of an edible part, the mesocarp, which is consumed by the local population. It represents 20% of the fruit and is conventionally used for its nutritional and therapeutic qualities. Doum mesocarp (DM) is rich in phytochemical ingredients such as flavonoids, phenolic acids, and saponins, which exhibit antioxidants, anti-inflammatory, and lipid-lowering effects (15, 16). It has been demonstrated that DM’s anti-inflammatory and antioxidant qualities alter lipid metabolism (13) and protect against oxidative stress-induced damage to the cardiovascular and renal systems (6). The flavonoids and phenols of the methanol extract of DM control lipid profile and hypertension by lowering the angiotensin-converting enzyme (6).

Doum fruit is deemed one of the fruits that generate the most waste since the inedible portion (endosperm or seed) makes up over 80% of the fruit (17). This waste is difficult to dispose of because of landfill space constraints, environmental regulations, and disposal costs, especially in the nations where Doum was discovered (18). Although little is known about the doum inedible endosperm (DE), its physicochemical properties indicate beneficiation (14). The inedible DE could be considered the richest source of polysaccharides, particularly mannan (19). Research on the bioactive constituents and medical applications of inedible DE is limited, and the potential medical benefits have not been explored. More studies are required to confirm its safety and reliability. Besides, the edible part of Doum (DM) needs confirmatory data.

Additionally, the mechanism of dyslipidemia and the type of blood lipid profile that develops in chronic kidney disease (CKD) remain under investigation. Under these circumstances, the existing work is conducted to assess the potential protective effect of DM and DE (for the first time) aqueous extracts in two doses of hyperlipidemia and its complications as a risk factor for CVDs and renal dysfunction in experimental Wistar rats. The present investigation will take into consideration the identification of phenols, flavonoids, and phytochemical screening of DM and DE (for the first time) to explain to what extent these bioactive constituents are correlated to their benefits on the blood lipid profile, cardiovascular risk, including atherosclerosis index, angiotensin-converting enzyme, an indicator of hypertension, and renal dysfunction.

## 2 Materials and methods

### 2.1 Materials

Atorvastatin (commercially available as Atorlip®, in the form of calcium trihydrate equivalent to 10 mg of atorvastatin) was purchased from a local pharmacy in Qassim Province (Globalpharma Co. LLC, P. O. Box 72,168, Dubai, UAE).

Triton X-100, a non-ionic detergent of laboratory grade, was purchased from Sigma-Aldrich (Merck, Germany; CAS No. 92046–34-9).

Commercial colorimetric kits for laboratory use were purchased from Biodiagnostic Diagnostic and Research Reagents Company, Cairo, Egypt, including:

(1) lipid profile detection: triglyceride (TG; catalogue no. TR 20 30), total cholesterol (TC; catalogue no. CH 12 20), and high-density lipoprotein (HDL-C; catalogue no. CH 12 30). (2) Renal function markers: Creatinine, urea, and uric acid as indicators of kidney function with catalogue numbers CR 12 51, UR 21 10, and UA 21 20, respectively. (3) Antioxidant enzyme activity: glutathione peroxidase (GHPx) and superoxide dismutase (SOD) in kidney tissue with catalog numbers GP 25 26 and SD 25, 23, respectively. (4) lipid peroxidation: Malondialdehyde (MDA) was detected by calorimetry with catalog number MD 25 31.

On the other hand, commercial ELISA kits were obtained from Elabscience, USA, including:

(1) Enzyme catalase antioxidant activity (CAT) detection, with catalog number (CAT; cat. no. M-BC-K031-S).
(2) Angiotensin-converting enzyme (ACE) measurement, with catalog number (E-EL-R2401), and a coefficient of variation <10%.

### 2.2 Animals

Healthy adult male Wistar rats (7 weeks old, weighing 180–200 g) were obtained from the King Saud University Laboratory Animal Centre in Riyadh, Saudi Arabia. The animals were transferred to a suitable room for rearing at the Department of Food Science and Human Nutrition, College of Agriculture and Food, Qassim University, Buraydah, Saudi Arabia. Before commencing the experiment, the animals were acclimated to the experimental room by housing them in wire cages (five per cage) for 1 week. The raising room was set up for optimal conditions, including a photo period (12 hrs of light and 12 hrs of dark cycle), a temperature of 23 ± 2 °C, and a relative humidity of 51 ± 5%. Throughout the trial, the animals were provided with fresh water and a basal commercial diet *ad libitum*. The General Company of Feed Mills supplied the commercial diet, which provided the nutrient requirements recommended by the National Research Council (20)complied with the animal care guidelines The research experiment complied with the animal care guidelines suggested by Qassim University’s Deanship of Scientific Research and followed the ‘International Animal Ethics Committee’. The study was reported in accordance with the 10 ARRIVE guidelines. This work was approved by Qassim University Health Research Ethics Committee, Kingdom of Saudi Arabia, approval number “23–61-07”.

### 2.3 Acute Oral toxicity test

A traditional method for assessing the acute toxicity of the extract was used with certain modifications, particularly for DE, which had not been previously studied, to ensure the selected doses were non-toxic. The acute oral toxicity test was performed in accordance with OECD Guideline No. 423 (21). The rats were divided into six groups, with three animals per group. They were subjected to aqueous extracts of DM or DE. The first three groups received DM, the other groups were offered DE at different doses (50, 500, and 1,000 mg/kg/day) orally for 2 weeks, respectively. The rats were observed daily for weakness, illness symptoms, or even death. Since there was no sign of toxicity, the dose was increased to 2000, 2,500, and 3,000 mg/kg. The final finding indicated that DM or DE aqueous extract was harmless up to 3,000 mg/kg body weight (BW) when administered orally. The two doses of DM and DE selected for the present study were 500 and 1,000 mg/kg.

### 2.4 Hyperlipidemia induction

On day 21 of the experiment, a single intraperitoneal injection (i/p) of TrX-100 at a dose of 100 mg/kg BW was used to induce hyperlipidemia (22). A booster dose of TrX-100 was administered intraperitoneally on day 30 to ensure sustained hyperlipidemia throughout the experiment (23).

### 2.5 *Hyphaene thebaica* (Doum palm)

*Hyphaene thebaica* L. fruit was obtained from the local market area, where dates and other public products are sold, in Qassim Province, KSA. The fruit species was recognized and validated at Qassim University’s Department of Botany, College of Agriculture. *Hyphaene thebaica,* family Palmae, is indexed in Herbarium Global Plants under the barcode E00349917 and cultivated in West Asia and Egypt.

### 2.6 Preparation of the DM and DE aqueous extracts

Fifty fresh *H. thebaica* fruits, free of physical flaws, were chosen randomly and repeatedly cleaned with tap water. The endocarp (inner inedible section) and the mesocarp (outer edible part) of *H. thebaica* fruits were separated using stainless steel knives. The endocarp was cracked with a hammer, and the inner part was obtained, which was cleaned to get the seed (endosperm). The mesocarp and endosperm were milled separately into a fine powder using an electric stainless-steel grinder (SF5668CG, China). To remove any chance of microbial growth, the finely ground mesocarp and endosperm powder were dehydrated in an oven set at 50 °C until two consecutive consistent weights were obtained (14). The fruit was crushed and then soaked in deionized water at a 5:1 ratio v/w to prepare aqueous extracts of DM and DE. This process was designed to produce a high concentration of flavonoids and total phenolic components. The extraction process was performed for 12 h at an average temperature of 22 ± 2 °C (24). The crude extract was calculated for the mesocarp and endosperm, which were 24.3 and 10.7%, respectively. The crude extract yield was calculated as follows:


$$\text{Yield}\%=\frac{\text{Weight of extract}\;(g)}{\text{Weight of sample}\;(g)}$$

The crude extract was diluted with distilled water to a concentration of 100 mg/mL to facilitate preparation of the chosen doses, 500 and 1,000 mg/kg.

### 2.7 DM and DE total phenolic and total flavonoid constituents

Conditions for a UV/Vis spectrophotometer (Jenway, England) were 20 °C and 37% relative humidity (RH). Total phenols were determined using colorimetry with the Folin–Ciocalteu reagent, based on the regression equation of the standard plot (y = 1001.1x + 4.4832, *r*^2^ = 0.9993) and expressed as milligrams of gallic acid equivalent per kilogram of sample (25). The colorimetric aluminum chloride method was employed to ascertain the total flavonoid content (26). One milliliter of the sample extract, three milliliters of methanol, 0.2 milliliters of 10% aluminum chloride, 0.2 milliliters of potassium acetate (1 M), and 5.6 milliliters of distilled water were added. After that, the mixture was left to rest for 30 min at room temperature. The absorbance was measured at 420 nm. One mg/ml of rutin was chosen as a standard dosage. Using the usual plot regression equation (y = 372.82x–4.2562, *r*^2^ = 0.996), the total flavonoid content was calculated as milligrams of return equivalent per 100 grams in three samples.

### 2.8 DM and DE antioxidant activities

An assay method by (27) and a UV/Vis Spectrophotometer (Jenmy, England) were employed to measure 2,2-diphenyl-1-picrylhydrazyl (DPPH) at 24 °C and 32.3% relative humidity (RH). Methanol was used to prepare concentrations ranging from 2/100 g to 10/100 g from a sample. DPPH radical (100 μL, 0.2 mM) and extract (100 μL) were mixed with methanol. The mixture was stirred and left for 15 min in a dark place. After that, the absorbance at 517 nm was measured and compared to a blank. At 25 °C and 38% relative humidity, the test was performed. The percentage scavenging effect was calculated as [(Ao - A1) /Ao] x 100, since Ao represents the absorbance of the blank and A1 represents the sample reading.

### 2.9 DM and DE phytochemical screening (high-performance liquid chromatography, HPLC)

The procedure was performed according to the Agilent Application Note (Publication 5,991-3801EN, 2014). Kinetex® 1.7 μm EVO C18 50 mm x 2.1 mm column (Phenomenex, United States) was employed alongside an Agilent 1,260 Infinity HPLC Series (Agilent, USA) and a quaternary pump. The utilized temperature was 30 °C. Using (1) HPLC grade water with 0.1% H3PO4 (v/v), (2) acetonitrile with 0.1% H3PO4 (v/v), and (3) a methanol flow rate of 0.2 mL/min, a ternary linear elution gradient was employed for dissociation. Twenty microliters were injected. Detection: At 20 °C and 38% relative humidity (RH), using a wavelength of 280 nm. Phenolic compound peaks were identified by matching their retention times and UV spectra to reference standards.

### 2.10 Atorvastatin as a standard drug

Atorvastatin was used as a control reference balance due to its hypolipidemic effect. The dose of atorvastatin offered was 8 mg per kg BW daily, suspended in distilled water; this dose was calculated according to the corresponding therapeutic dose recommended for humans, which ranges from 20 to 80 mg following the conversion equation previously suggested by Shin et al. (28).

### 2.11 Protocol and design of the experiment

Seventy rats were weighed separately and randomly assigned to seven experimental groups (*n* = 10 per group), using a computer-generated random number table to ensure unbiased group allocation. The experimental groups were designed as follows: Group 1: negative control, received a basal diet and gavage with saline solution throughout the experimental period. Group 2: positive control received the same treatment as Group 1. Group 3: the standard drug control was gavage with atorvastatin standard drug, 8 mg/kg BW daily. Groups 4 and 5 were offered doum mesocarp aqueous extract (DM) at 500 mg/kg BW and 1,000 mg/kg BW daily, respectively (29). Groups 6 and 7 were gavaged with doum endosperm aqueous extract (DE) at 500 mg/kg BW/day and 1,000 mg/kg BW/day, respectively. It means that pretreatment with Doum extracts (DM and DE) and atorvastatin began on day 1 and continued for 40 days. On day 21 of the experiment, all the groups except the negative control group were injected intraperitoneally (i/p) with 100 mg/kg BW of TrX-100 as a single dose to induce a hyperlipidemic rat (30). A booster dose of TrX-100 (100 mg/kg BW) was administered i/p on day 30 to all groups except the negative one to ensure sustained hyperlipidemia (23). This staggered design allows assessing the ability of the extracts to both prevent the onset of dyslipidemia and its complications during the first 21 days, and to modulate established hyperlipidemia and associated organ damage during the subsequent 19 days. Group (2), injected with TrX-100 without supplementation, was a positive control (model). The experimental design was summarized in Table 1. To adjust the supplemental dosage, the rats were weighed once a week. On day 40, blood samples were collected via the retro-orbital plexus under light diethyl ether anesthesia, performed by a trained technician to minimize distress. All procedures adhered to the approved animal ethics protocol by Qassim University Health Research Ethics Committee, Kingdom of Saudi Arabia, approval number “No. 23–61-07” and followed the AVMA guidelines for euthanasia. Serum was extracted and kept in a deep freezer at −20 °C for biochemical analysis after blood was centrifuged for 10 min at 2000 × g. Eight samples were used for biochemical analysis, including lipid profile (TG, TC, and HDL-C) levels and kidney function tests (creatinine, urea, and uric acid). Three animals from each group were randomly selected, and euthanasia was performed to reduce the pain. The rats were anesthetized with diethyl ether at a concentration of 1.9% (0.08 mL per liter of volume of a container used) before being sacrificed according to AVMA Guidelines for Euthanasia in Animals. The two kidneys were carefully handled, rinsed in chilled 1.15% potassium chloride (KCl), and then placed on filter paper. The left kidney was weighed, then five samples were assessed for lipid peroxidation, MDA, and antioxidant enzyme activity (GHPx, SOD, and CAT) in the kidney tissue. Three samples from the right one were kept for histological examination.

**Table 1:** Experimental design.

Groups	Days 1–40	Day 21 TrX-100 i/p injection (100 mg/kg)	Day 30 booster dose of TrX-100 i/p injection (100 mg/kg)
(1) Control negative	Basal diet without supplementation		
(2) Control positive (model)	Basal diet without supplementation	√	√
(3) Control a drug standard	Atorvastatin 8 mg/kg BW	√	√
(4) Treatment group	Mesocarp extract 500 mg/kg BW	√	√
(5) Treatment group	Mesocarp extract 1,000 mg/kg BW	√	√
(6) Treatment group	Endosperm extract 500 mg/kg BW	√	√
(7) Treatment group	Endosperm extract 1,000 mg/kg BW	√	√

### 2.12 Relative kidney weights

A sensitive balance was used to determine the absolute weight of the left kidney, after it had been cleansed in a standard saline solution. The relative kidney weight was calculated as the ratio of kidney weight to body weight. The relative kidney weight was calculated by dividing the absolute kidney weight by the body weight (16).

### 2.13 Kidney homogenization

The left kidneys were homogenized according to the manufacturer’s instructions for the respective assay kits. Concisely, the kidney specimens were treated with phosphate buffer solution (pH 7.4) and then centrifuged at 1200 × g for 30 min at 4 °C to obtain the supernatant fluid. The protein concentration of the supernatant was determined according to the method of Lowry et al. (31). The collected supernatant was stored for quantifying antioxidant enzyme activities and lipid peroxidation.

### 2.14 Lipid profile and CVD risk indices

Serum lipid profile, including TG, TC, and HDL-C levels, was detected calorimetrically using commercial laboratory kits. Whilst low-density lipoprotein (LDL-C), very low-density lipoproteins (VLDL), total lipids (TLs), cardiac index (CI), coronary artery index (CAI), and atherogenic index (AI) were calculated (32–35):


LDL−C(mgdL)=Total cholesterol−HDL−Triglycerides/5

VLDL conc.(mgdL)=Triglycerides/5

TLs,(mgdL)=(2.27xTotal cholesterol)+TG+62.3

CAI=(LDL−chol)/(HDL−chol)

CI=(Total cholesterol)/(HDL−chol)

AI=(Total cholesterol−HDL−chol)/(HDL−chol)

### 2.15 Angiotensin-converting enzyme

A commercial ELISA kit was used to determine ACE as an indicator of hypertension (mentioned in the materials section).

### 2.16 Kidney function tests

Serum creatinine, urea, and uric acid levels were measured using colorimetric kits as indicators of kidney function (mentioned in the materials section).

### 2.17 Nephroprotection percentage

According to the formula suggested by Syed et al. (36), the percentages of nephroprotection (NP%) of groups receiving 500 and 1,000 mg DM and DE were determined for each biochemical parameter (creatinine, urea, and uric acid) independently:


$$\mathrm{NP}\%=[1-\frac{\left(T-N\right)}{P-N}]\times 100$$

T = treatment means, P = positive means, and N = negative means. The total nephroprotection percentage (TNP%) was compared to the negative group, assuming it was 100%.

### 2.18 Kidney antioxidant activities and lipid peroxidation

Commercial colorimetric kits were used to measure the antioxidant enzyme activities of GHPx and SOD concentration in kidney tissue. Meanwhile, the CAT antioxidant activity was measured by ELISA kits. MDA was measured calorimetrically using kits as an indicator of lipid peroxidation (mentioned in the materials section).

### 2.19 Histopathological observation

The right kidney specimens, which had been previously stored for observing histological architecture, were preserved in formal saline at a concentration of 10% and treated with standard paraffin wax. The kidney sections were examined for architectural changes. The tissues were stained with Hematoxylin and Eosin (H and E) (37).

### 2.20 Statistical analysis

Data are presented as means ± standard error (SE). A one-way analysis of variance (ANOVA) was conducted for each measured parameter using SAS version 20 (SAS Institute, USA) to compare differences among the seven experimental groups, which were treated as fixed effects. When ANOVA indicated significant overall differences (*p* < 0.05), Tukey’s Honestly Significant Difference (HSD) *post hoc* test was applied to compare the negative control group with other experimental groups and the positive control (model) group with the treated groups.

The normality of residuals was assessed using the Shapiro–Wilk test, and homogeneity of variances was evaluated using Levene’s test. All datasets met the assumptions of normality and homoscedasticity (*p* > 0.05). For parameters where assumptions were not fully met, non-parametric alternatives, such as the Kruskal-Wallis test followed by Dunn’s *post hoc* test, were considered; however, all reported results are based on parametric tests, as the assumptions of normality and homogeneity of variance were satisfied. The statistical model applied was:


Yij=μ+Ti+εij

Where $Y_{\mathrm{ij}}$ is the observed value for the animal j in treatment i , μ is the overall mean, $T_{i}$ is the fixed effect of treatment i , and $\varepsilon_{\mathrm{ij}}$ is the random error term.

## 3 Results

### 3.1 DM and DE total phenols, total flavonoids, antioxidant activity, and phytochemical screening

The data in Table 2 indicate that DM is rich in total phenolic and flavonoid compounds (1551.80 ± 78.6 and 176.45 ± 28.7, respectively) and exhibits strong antioxidant activity, as measured by DPPH radical-scavenging activity at concentrations of 0.10, 0.25, and 0.50% (46.89 ± 9.8, 90.11 ± 15.8, and 98.20 ± 12.6, respectively). Phytochemical screening of DM revealed the presence of multiple bioactive compounds, including catechin, catechol, P-hydroxybenzoic acid, vanillic acid, rutin, ferulic acid, chlorogenic acid, syringic acid, resveratrol, quercetin, P coumaric acid, gallic acid, and kaempferol, as identified by HPLC analysis (Table 3). In contrast, DE exhibited lower levels of total flavonoids, total phenols, and antioxidants (Table 2), as well as fewer phytochemical constituents compared to DM (Table 4). To the best of our knowledge, the phytochemical composition of DE has not been previously studied.

**Table 2:** Total phenols, flavonoids, and antioxidant activity of Doum mesocarp (DM) and Doum endosperm (DE)°.

Doum	Total phenols (mg gallic acid equivalent/kg)	Total flavonoids (mg rutin equivalent/100 g)	% DPPH Radical-Scavenging Activity		
			0.10%	0.25%	0.50%
DM	1551.80 ± 78.6	176.45 ± 28.7	46.89 ± 9.8	90.11 ± 15.8	98.20 ± 12.6
DE	17.71 ± 6.5	0.92 ± 0.05	2.19 ± 0.7	6.60 ± 1.8	10.99 ± 3.7

DPPH: 2,2-Diphenyl-1-picrylhydrazyl is expressed as a percentage inhibition. Mean± Standard error (SE). °Sample size: 3.

**Table 3:** Bioactive phytochemical constituents screening of DM detected by HPLC.

Name	Expected retention time	Retention time (min)	Area	Amount (mg/kg) °
Gallic	3.450	3.40	13.8868	1.380
Catechol	4.500	4.49	28.8503	9.501
P hydroxybenzoic	6.550	6.50	20.0406	5.104
Catechin	7.000	6.88	20.4534	9.935
Chlorogenic acid	7.600	7.66	8.7633	2.738
Vanillic acid	8.000	8.12	30.1138	5.000
Caffeic acid	8.400	8.46	8.4172	0.979
Syringic acid	9.400	9.35	22.6126	2.154
P Coumatic	10.650	10.63	22.6955	1.681
Rutin	12.200	12.1	29.3933	4.232
Ferulic	12.600	12.47	7.4188	2.810
OCumaric	13.850	13.88	12.1597	0.716
Hesperidin	14.700	14.64	4.7465	0.957
Resveratrol	16.600	16.53	6.1407	1.782
Myricetin	17.500	17.80	4.3983	0.525
Quercetin	18.800	18.71	1.6392	1.747
Apigenin	20.000	19.98	2.4529	0.037
Kaempferol	20.100	20.23	7.3480	1.027

**Table 4:** Bioactive phytochemical constituents screening of DE detected by HPLC.

Name	Expected retention time	Retention Time (min)	Area	Amount (mg/kg) °
Gallic	3.400	3.39	2.4596	0.122
P hydroxybenzoic	6.550	6.48	2.0625	0.263
Catechin	7.000	6.86	1.8838	0.458
Chlorogenic acid	7.600	7.63	2.9674	0.464
Vanillic acid	8.000	7.93	3.2091	0.266
Caffeic acid	8.400	8.28	3.4258	0.199
Syringic acid	9.400	9.36	1.6908	0.081
P Coumatic	10.650	10.63	1.8989	0.070
Rutin	12.200	12.08	4.7779	0.344
Ferulic	12.600	12.48	1.4226	0.269
Ocumaric	13.850	13.89	1.6900	0.050
Resveratrol	16.600	16.54	6.1625	0.894
Myricetin	17.500	17.72	2.0306	0.121
Apigenin	20.000	19.74	1.9779	0.015
Kaempferol	20.100	20.23	7.6158	0.539

To make reading easier, the standard error of the mean, which did not surpass 10% of the mean value, was eliminated from the mean of three separate plant samples taken from the exact location. DM: Doum Mesocarp.

To make reading easier, the standard error of the mean, which did not surpass 10% of the mean value, was eliminated from the mean of three separate plant samples taken from the exact location. DE: Doum Endosperm.

### 3.2 Effect of DM and DE on body gain and kidney weight (absolute and relative)

The negative control group exhibited a body weight gain of 84.6 ± 3.1 g. The TrX-100 model group showed a significantly (*p* < 0.01) higher BW gain, BW gain %, and relative kidney weight of 108.2 ± 2.3 g, 59.5 ± 1.3, and 0.58 ± 0.016 g, respectively (Table 5). Atorvastatin treatment significantly (*p* < 0.05) reduced BW gain, BW gain %, and relative kidney weight (91.5 ± 3.1, 45.8 ± 3.5, and 0.43 ± 0.028 g), respectively. Supplementation of DM at 500 mg/kg and 1,000 mg/kg significantly reduced BW gain (89.1 ± 3.9 g and 95.3 ± 2.6 g, *p* < 0.05), BW % (47.6 ± 3.1% and 47.7 ± 2.3%, p < 0.05), and relative kidney weight (0.43 ± 0.036 and 0.42 ± 0.027 g, *p* < 0.05), respectively. DE at 1000 mg/kg also significantly lowered BW gain, BW %, and relative kidney weight (*p* < 0.01), compared to the TrX-100 model group. Similar results were observed with DE at 500 mg/kg, with a significance level of p < 0.05 for relative kidney weight.

**Table 5:** Effect of Doum mesocarp and endosperm on body gain, absolute and relative kidney weight °.

Group	Initial BW (g)	Final BW (g)	BW gain (g)	BW gain %	Absolute kidney wt. (g)	Relative kidney wt.
Saline negative control	197.5 ± 2.4	281.4 ± 4.8	84.6 ± 3.1	42.4 ± 1.8	1.17 ± 0.16	0.41 ± 0.025
Tr positive control (model)	181.8 ± 3.1	289.4 ± 9.5	108.2 ± 2.3**	59.3 ± 1.9**	1.68 ± 0.18	0.58 ± 0.016**
Tr + Atorvastatin	200.0 ± 4.2	291.8 ± 5.8	91.5 ± 3.1^a^	45.5 ± 3.5^a^	1.28 ± 0.08	0.43 ± 0.028a
Tr + DM 500 mg/kg	187.2 ± 3.6	276.8 ± 5.2	89.1 ± 3.9^a^	47.2 ± 3.1^a^	1.21 ± 0.14	0.43 ± 0.036^a^
Tr + DM 1000 mg/kg	199.7 ± 5.2	294.5 ± 7.3	95.3 ± 2.6^a^	47.9 ± 2.5^a^	1.26 ± 0.21	0.42 ± 0.027^a^
Tr + DE 500 mg/kg	198.3 ± 5.7	286.1 ± 6.9	87.1 ± 3.2^b^	43.4 ± 1.7^b^	1.15 ± 0.09	0.40 ± 0.038^a^
Tr + DE 1000 mg/kg	189.7 ± 4.9	270.1 ± 7.5	80.8 ± 2.7^b^	42.2 ± 1.6^b^	1.17 ± 0.19	0.43 ± 0.022^b^
*p*-value	*p* = 0.146	*p* = 0.128	*p* = 0.039	*p* = 0.041	*p* = 0.148	*p* = 0.029

Means ± standard error (SE). The means of the Tr positive control in the same column have the sign**, significantly different from the saline negative control at *p* < 0.01. The means of the treated groups have subscripts letters a or b, significantly different from the Tr positive control at *p* < 0.05 and *p* < 0.01, respectively. Tr: TrX-100; DM: Doum mesocarp; DE: Doum endosperm. Sample size: 8.

### 3.3 Effect of DM and DE on lipid profile

The TrX-100 model control group showed significant elevation (*p* < 0.01) of TGs, TC, LDL-C, and TLs levels (124.2 ± 9.7, 130.2 ± 7.4, 81.4 ± 5.3, and 481.4 ± 24.8 mg/dL), respectively, whereas the elevation of VLDL was significant at (*p* < 0.05; Table 6). Administration of atorvastatin significantly reduced TGs, TC, and LDL-C compared to the TrX-100 model control. Administration of DM at 1000 mg/kg showed hypolipidemia, which manifested by significantly (*p* < 0.01) lowered TGs, TC, and LDL-C (68.8 ± 3.4, 80.1 ± 3.5, and 31.5 ± 5.4) mg/dl, respectively, and TLs at (*p* < 0.05) while increasing HDL-C (35.6 ± 3.3 mg/dL) as compared with the model group. The same hypolipidemic effect was observed with supplementation of DM at 500 mg/kg, but it was insignificant. A 500 mg/kg BW DE showed a significant reduction in TGs (*p* < 0.05), TC, LDL-C, and TLs (p < 0.01). It appears that both 1,000 mg/kg DM and 500 mg /kg DE significantly ameliorated surrogate indices of cardiovascular risk (CAI, CI, AI, ACE), restoring values closer to those of the normal control group. Meanwhile, DE 1000 mg/kg was less pronounced; it recorded a significant decrease (*p* < 0.05) in TC, LDL-C only.

**Table 6:** Effect of Doum mesocarp and endosperm on lipid profile (triglyceride, total cholesterol, HDL-C, LDL-C, Total lipid, and VLDL).

Group	TGs mg/dL	TC mg/dL	HDL-C mg/dL	LDL-C mg/dL	TLs mg/dL	VLDL mg/dL
Saline negative control	64.4 ± 2.4	75.4 ± 2.8	33.8 ± 2.6	29.6 ± 1.9	298.5 ± 16.6	13.2 ± 1.9
Tr positive control (model)	124.2 ± 9.7**	130.2 ± 7.4**	23.4 ± 3.8	81.4 ± 5.3**	481.4 ± 24.8**	25.6 ± 2.6*
Tr + Atorvastatin	90.6 ± 3.7^a^	101.6 ± 4.6^a^	32.0 ± 2.4	51.9 ± 3.5^a^	382.6 ± 19.4	19.0 ± 2.8
Tr + DM 500 mg/kg	110.7 ± 5.2	115.8 ± 4.2	30.4 ± 2.7	62.1 ± 4.6	434.9 ± 21.8	22.8 ± 1.4
Tr + DM 1000 mg/kg	68.8 ± 3.4^b^	80.1 ± 3.5^b^	35.6 ± 3.3	31.5 ± 5.4^b^	313.6 ± 25.2^a^	14.1 ± 3.1
Tr + DE 500 mg/kg	70.5 ± 6.8^a^	79.8 ± 5.7^b^	34.5 ± 2.7	32.1 ± 4.8^b^	314.5 ± 27.2^b^	14.8 ± 3.3
Tr + DE 1000 mg/kg	78.8 ± 8.9	86.6 ± 7.8^a^	32.4 ± 3.9	39.3 ± 5.9^a^	337.5 ± 24.8^a^	16.3 ± 2.6
*p*-value	*P* = 0.039	*p* = 0.030	*p* = 0.164	*p* = 0.035	*p* = 0.033	*p* = 0.049

Means ± standard error (SE). The means of the Tr positive control in the same column have the sign* or **, significantly different from the saline negative control at *p* < 0.05 and *p* < 0.01, respectively. The means of the treated groups have subscripts letters a or b, significantly different from the Tr positive control at *p* < 0.05 and *p* < 0.01, respectively. Tr: TrX100; DM: Doum mesocarp; DE: Doum endosperm. Sample size: 8.

### 3.4 Effect of DM and DE on mitigating CVD risk indices (CAI, CI, AI) and ACE

Table 7 revealed that the TrX-100 model rats had an increase in the risk of cardiovascular disorders, evidenced by significantly (*p* < 0.05) elevated values for CAI, CI, AI, and ACE (3.36 ± 0.57, 5.46 ± 0.68, 4.43 ± 0.43, and 20.26 ± 0.72) versus the negative control, respectively. The groups subjected to atorvastatin or low-dose DM showed a slight reduction in CAI, CI, AI, and ACE compared to the TrX-100 model. Meanwhile, groups treated with DM at 1000 mg/kg or DE at 500 mg/kg had significant improvements (*p* < 0.05) in CAI, CI, AI, and ACE, returning to near-normal levels. The effect of DE at 1000 mg/kg was less noticeable; it only significantly reduced AI (1.79 ± 0.71, *p* < 0.05), while not affecting CAI or CI.

**Table 7:** Effect of Doum mesocarp and endosperm on mitigating cardiovascular risk indices (coronary artery index, cardiac index, and atherogenic index) and angiotensin-converting enzyme.

Group	LDL/HDL CAI	TC/HDL CI	AI	ACE ng/ml
Saline negative control	0.87 ± 0.38	2.21 ± 0.52	1.24 ± 0.45	15.68 ± 0.94
Tr positive control	3.36 ± 0.57*	5.46 ± 0.68*	4.43 ± 0.43*	20.26 ± 0.72*
Tr + Atorvastatin	1.68 ± 0.43	3.23 ± 1.12	2.22 ± 0.76	17.51 ± 1.05
Tr + DM 500 mg/kg	2.10 ± 0.68	3.71 ± 1.28	3.08 ± 1.05	18.56 ± 0.97
Tr + DM 1000 mg/kg	0.87 ± 0.18^a^	2.37 ± 0.55^a^	1.36 ± 0.48^a^	16.18 ± 0.83^a^
Tr + DE 500 mg/kg	0.89 ± 0.15^a^	2.28 ± 0.63^a^	1.38 ± 0.47^a^	16.02 ± 0.87^a^
Tr + DE 1000 mg/kg	1.18 ± 0.94	2.55 ± 0.96	1.79 ± 0.71^a^	18.15 ± 1.18
*p*-value	*p* = 0.008	*p* = 0.009	*p* = 0.010	*p* = 0.048

Means ± standard error (SE). The means of the Tr positive control in the same column have the sign*, significantly different from the saline negative control at *p* < 0.05. The means of the treated groups have subscripts letter a, significantly different from the Tr positive control at *p* < 0.05. Tr: TrX-100; DM: Doum mesocarp; DE: Doum endosperm; Coronary artery index (CAI) = LDL-chol/HDL-chol, Cardiac index (CI) = TC/HDL-chol; and Atherogenic index (AI) = (total cholesterol–HDL-chol) / HDL-chol; ACE: Angiotensin-converting enzyme. Sample size: 8. Sample size: 5.

### 3.5 Effect of DM and DE on kidney function (creatinine, urea, and uric acid)

As shown in Table 8, the TrX-100 model control induced kidney dysfunction, evidenced by a significant increase in creatinine, urea, and uric acid levels (*p* < 0.05; 1.57 ± 0.24, 48.43 ± 2.4, and 3.87 ± 0.14) mg/dl, respectively, comparable to the negative group. The nephroprotective effect was observed in the groups treated with atorvastatin (standard drug) and 1,000 mg/kg DM, indicated by a significant (*p* < 0.05) reduction in creatinine, urea, and uric acid levels compared to the TrX-100 model group. At the same time, DM at 500 mg/kg, DE at two doses (500 and 1,000 mg/kg) showed mild nephroprotective effects. While they each recorded a significant (*p* < 0.05) decrease in creatinine levels, they had no influence on urea or uric acid levels.

**Table 8:** Effect of Doum mesocarp and endosperm on kidney function (creatinine, urea, and uric acid) °.

Group	Creatinine Mg/dl	Urea Mg/dl	Uric acid Mg/dl
Saline negative control	0.56 ± 0.08	24.28 ± 2.6	2.15 ± 0.12
Tr positive control	1.57 ± 0.24*	48.43 ± 2.4*	3.87 ± 0.14*
Tr + Atorvastatin	0.48 ± 0.07^a^	28.47 ± 2.1^a^	2.67 ± 0.18^a^
Tr + DM 500 mg/kg	0.46 ± 0.19^a^	44.71 ± 4.2	3.27 ± 0.15
Tr + DM 1000 mg/kg	0.47 ± 0.07^a^	31.56 ± 2.3^a^	2.52 ± 0.16^a^
Tr + DE 500 mg/kg	0.61 ± 0.11^a^	40.72 ± 2.7	3.16 ± 0.26
Tr + DE 1000 mg/kg	0.52 ± 0.18^a^	43.94 ± 4.5	2.95 ± 0.29
*p*-value	*p* = 0.007	*p* = 0.042	*p* = 0.009

Means ± standard error (SE). The means of the Tr positive control in the same column have the sign*, significantly different from the saline negative control at *p* < 0.05. The means of the treated groups have subscripts letter a, significantly different from the Tr positive control at *p* < 0.05. Tr: TritonX-100; DM: Doum mesocarp; DE: Doum endosperm. Sample size: 8.

### 3.6 Total nephroprotection percentage of DM and DE

**Figure 1:**
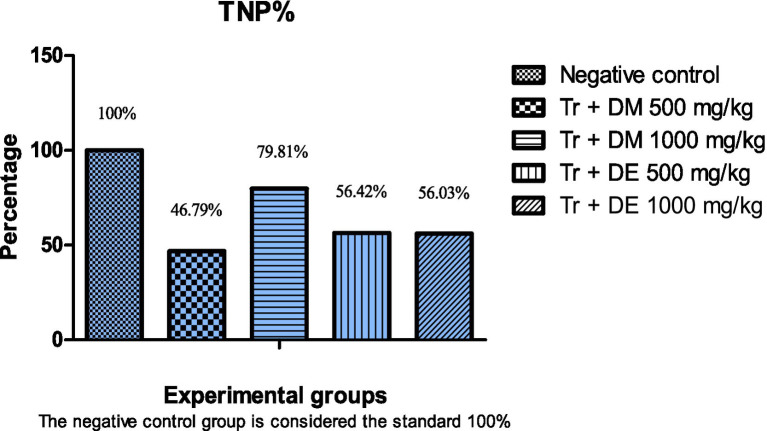
Effect of Doum mesocarp and endosperm on nephroprotection percentage. Tr: TritonX-100; DM: Doum mesocarp; DE: Doum endosperm; TNP%: Total nephroprotection percentage.

Analysis of the TNP% revealed that the high dose of DM (1,000 mg/kg) elicited a superior nephroprotective effect (79.81%) compared to other treatment groups (Figure 1). There was a slight variation in the TNP% between the groups supplemented with DM 500 mg/kg, DE 500 mg/kg, and DE 1000 mg/kg (46.79, 56.42, and 56.03), respectively. The data aligned with the improvements in kidney function markers observed in Table 8, further supporting the nephroprotective properties of DM at 1000 mg/kg.

### 3.7 Effect of DM and DE on antioxidant activities and malondialdehyde content in kidney tissue

The TrX-100 model group showed oxidative stress and kidney damage manifested by significantly (*p* < 0.05) reduced antioxidant activities, GHPx, SOD, and CAT (31.7 ± 2.41, 27.6 ± 2.15, and 44.3 ± 3.63) U/mg protein, respectively, and increased MDA levels (34.7 ± 2.74) nmol/g protein (Table 9). Treatment with atorvastatin and supplementation with DM (1,000 mg/kg) significantly (*p* < 0.05) enhanced the activity of the antioxidant enzymes GSH-Px, SOD, and CAT, while reducing MDA levels, demonstrating their antioxidant and protective efficacy. Administration of both DM and DE at 500 mg/kg significantly (*p* < 0.05) improved GHPx and MDA levels, though their impact on SOD and CAT was less pronounced. Interestingly, DE at a high dose (1,000 mg/kg) did not show improvement in the antioxidant activities of kidney tissue.

**Table 9:** Effect of Doum mesocarp and endosperm on antioxidant activities and malondialdehyde content in kidney tissue.

Group	Glutathione Peroxide U/mg protein	Superoxide Dismutase U/ mg protein	Catalase U/mg protein	malondialdehyde nmol/g protein
Saline negative control	62.5 ± 3.33	43.7 ± 3.37	69.7 ± 4 0.52	12.4 ± 1.57
Tr positive control	31.7 ± 2.41*	27.6 ± 2.15*	44.3 ± 3.63*	34.7 ± 2.74*
Tr + Atorvastatin	56.7 ± 3.64^a^	39.8 ± 2.56^a^	65.5 ± 4.71^a^	17.8 ± 1.84^a^
Tr + DM 500 mg/kg	49.1 ± 3.56^a^	35.8 ± 2.81	58.4 ± 4.58	19.6 ± 1.73^a^
Tr + DM 1000 mg/kg	51.7 ± 3.25^a^	38.8 ± 2.16^a^	63.8 ± 3.62^a^	20.4 ± 1.92^a^
Tr + DE 500 mg/kg	45.9 ± 3.43^a^	34.5 ± 3.72	56.2 ± 4.94	24.3 ± 1.37^a^
Tr + DE 1000 mg/kg	41.8 ± 4.75	36.8 ± 2.78	46.9 ± 4.36	26.1 ± 2.28
P-value	*p* = 0.049	*p* = 0.037	*p* = 0.025	*p* = 0.038

Means ± standard error (SE). The means of the Tr positive control in the same column have the sign*, significantly different from the saline negative control at *p* < 0.05. The means of the treated groups have subscripts letter a, significantly different from those of the Tr positive control at *p* < 0.05. Tr: TritonX-100; DM: Doum mesocarp; DE: Doum endosperm. Sample size: 5.

### 3.8 Histopathological examination of renal tissue

**Figure 2:**
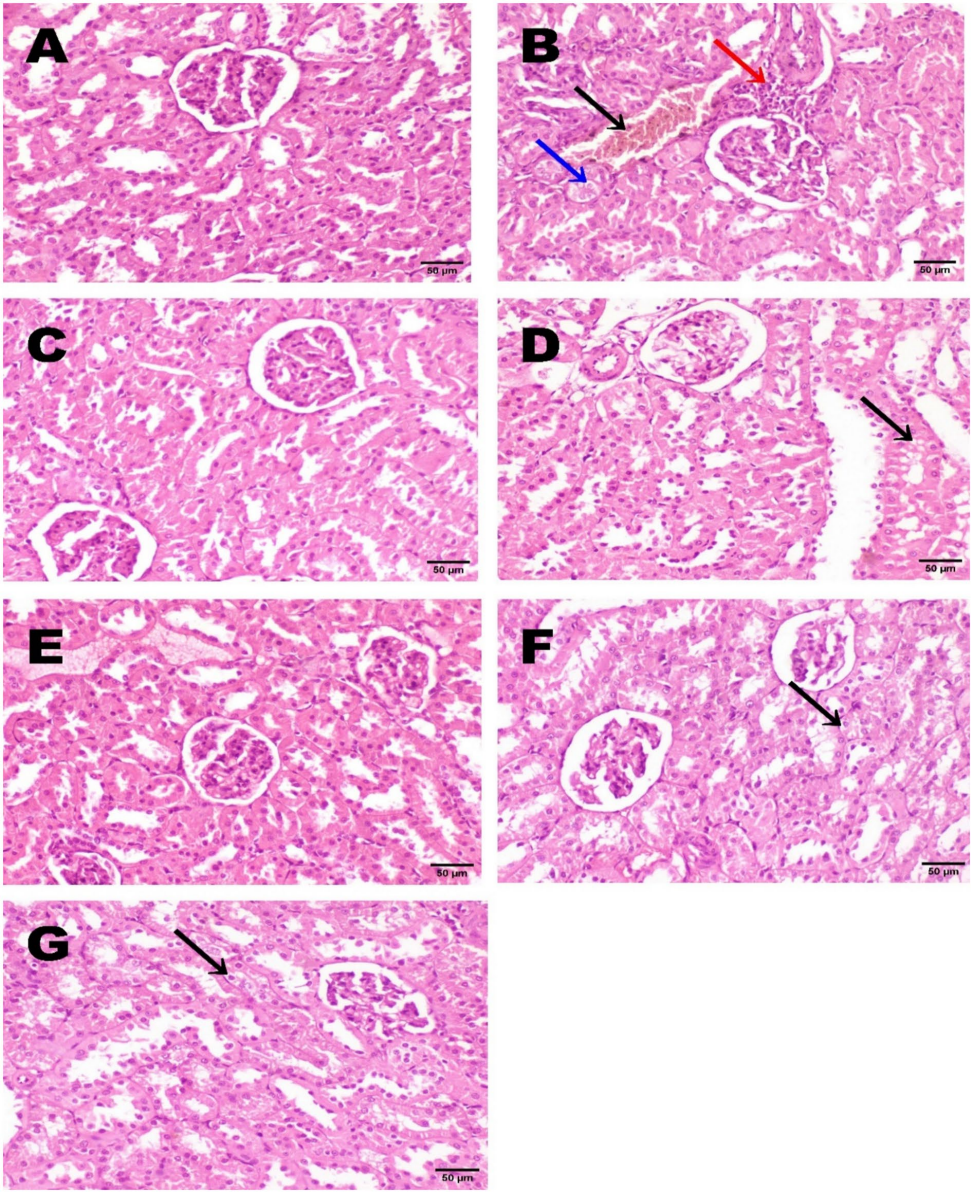
Histopathological observations of renal tissue in experimental groups. A photomicrograph of renal tissue shows the negative control of the normal histoarchitecture of renal parenchyma **(A)**. The TrX-100 model group exhibited congestion of renal blood vessels (black arrow), vacuolar degeneration of epithelial lining renal tubules (blue arrow), and perivascular inflammatory cell infiltration (red arrow) **(B)**. The renal section of the standard atorvastatin group illustrated normal histoarchitecture of renal parenchyma **(C)**. Renal tissue of rats administered DM 500 mg/kg revealed no histopathological damage except slight degeneration of the vacuolar epithelial lining of some renal tubules (black arrow) **(D)**. Rats administered DM 1000 mg/kg showed a typical histological structure of renal tissue **(E)**. The epithelial lining of some renal tubules displayed vacuolar degeneration in groups supplemented with DE 500 and 1,000 mg/kg (black arrow) **(F,G)**, respectively. Sample size: 3 (H&E staining, 400×, scale bar = 50 μm).

Microscopic observation revealed normal renal histoarchitecture in the negative control group (Figure 2A). In contrast, the renal tissue from the model rats exhibited congestion of renal blood vessels, degradation of vacuolated epithelial cells in the renal tubules, and infiltration of perivascular inflammatory cells (Figure 2B). The renal tissue of the group treated with the standard drug, atorvastatin, displayed normal histoarchitecture of renal parenchyma (Figure 2C). Rats supplemented with a low dose of DM showed slight degeneration of the epithelial lining, and some sections showed minimal histological damage (Figure 2D). Meanwhile, a high dose of DM demonstrated no histopathological damage in all histological sections (Figure 2E). On the other hand, the administration of DE in both low and high doses showed degeneration of the vacuolar epithelial lining of some renal tubules (Figures 2F,G).

## 4 Discussion

The increase in body weight gain observed in the TrX-100 model group is consistent with previous studies (22, 38) that used TrX-100 and Triton WR − 1,339, respectively. They concluded that body weight gain was significantly increased in the Triton model group, which induced hyperlipidemia and promoted fat deposition in adipose tissue and around internal organs. The significant decrease in body weight gain observed with atorvastatin treatment is consistent with previous reports (16). Statins may block HMG-CoA reductase, reducing cholesterol synthesis and lowering triglyceride (TG) levels, which can lead to hypolipidemia and subsequently reduced body weight gain (39). The significant reduction in body gain achieved with DM and DE supplementations, comparable to the model group, was consistent with previous work (40), which revealed that body weight was significantly reduced in the group treated with the extract of *H. thebaica*. Conversely, the aqueous extract of Doum increased body weight gain (16). The conflict may be due to the low dose of Doum used in their study (20, 41 mg/kg). The highly significant reduction in BW gain shown in rats supplemented with DE agrees with (41). The endosperm is considered one of the highest natural sources of mannan (19), which balances the gut microbiota (42). Supplementing with mannose in a high-fat meal enhanced glucose homeostasis and reduced weight gain (43). The substantial increase in relative kidney weight observed in TrX-100 model rats suggests the presence of glomerulosclerosis, nephropathy, and consequent renal damage, consistent with previous reports (44). Atorvastatin, DM, and DE administrations normalized relative kidney weight to a level equal to that of the negative control group.

One critical risk factor for the onset of CVDs is hyperlipidemia (3) and renal dysfunction (4), both of which contribute substantially to global morbidity and mortality, which represent 30% of global deaths (5). This study seeks to evaluate the edible and inedible parts of *Hyphaene thebaica* as a natural alternative solution that has a hypolipidemic effect through using various parameters: CAI, CI, AI, ACE, and renal dysfunction, including blood creatinine, urea, and uric acid, and renal protective percentage, in addition to histopathological examination of renal tissue. Notably, TrX-100 is an acute hyperlipidemia model and may not reflect chronic diet-induced dyslipidemia or long-term cardiometabolic disease. Therefore, to address this limitation, two doses of Triton X-100 were administered: an initial dose followed by a booster dose 9 days later to ensure sustainable hyperlipidemia throughout the experimental period.

Dyslipidemia recorded in the group injected with TrX-100 (model) was indicated by a significant elevation in TG, TC, LDL-C, TL, and VLDL accompanied by a nonsignificant increase in HDL-C, which was agreed with previously published data (30, 45) which showed that hyperlipidemia induced by TrX-100 owing to the cessation of the TG-rich lipoproteins clearance, and encourages the synthesis of hepatic cholesterol (46) and promotes the absorption of intestinal lipid (11)). The notable decrease in TC, TG, and LDL-C in the group receiving atorvastatin, along with HDL-C values close to those of the negative control, confirmed previous work (1, 13) that concluded statin treatment has an anti-hyperlipidemic effect by reducing liver production of LDL-C. Statins exert their hypolipidemic effect by inhibiting the enzyme HMG-CoA reductase, which reduces the production of mevalonic acid—a key precursor in cholesterol biosynthesis (39).

Lipid profiles showed that DM at a high dose (1,000 mg/kg) achieved the best hypolipidemia effect, manifested by a significant decrease in TG, TC, LDL-C, and TL. In addition, the values of HDL-C and VLDL are close to the control negative value, which parallels the previous work (29). Serum TC, TG, and TL levels were significantly reduced after a few days of Doum fruit supplementation (47). H. Thebacia supplementation significantly rebalanced the blood lipid profile (40, 48). The hypolipidemic effect of DM may result from reduced lipid absorption in the gastrointestinal tract, potentially due to the formation of complexes with bile acids and cholesterol in the gut, thereby lowering total cholesterol levels in the bloodstream (1). Nevertheless, DM supplementation at a low dose (500 mg/kg) did not significantly improve the lipid profile, indicating a dose-dependent effect. This finding may be attributed to the low dose of DM (500 mg/kg), which does not contain sufficient phytochemicals to reduce blood lipids. To our knowledge, no published studies have investigated the inedible portion of doum (DE) as a hypolipidemic agent; therefore, the present study is the first to evaluate DE for this purpose. Interestingly, DE had low levels of total phenols, flavonoids, and phytochemical constituents, as mentioned earlier in the HPLC analysis. It achieved hypolipidemic performance, evidenced by a significant decrease in TG, TC, LDL-C, and TLs. These findings suggest that DE employs a different mechanism from DM in inducing hypolipidemia, which may be attributed to the polysaccharide and mannan content, which is concentrated in the endosperm (seed) and has hypolipidemic effects (14, 19). Polysaccharides, such as mannan, may improve the blood lipid profile more effectively than statins, which inhibit the enzymes responsible for cholesterol production in the liver. Mice pretreated with various forms of mannan before the induction of hyperlipidemia showed a significant decrease in serum atherogenic LDL (49). Mannan can modulate gut microbiota, reduce intestinal lipid absorption, and influence hepatic cholesterol synthesis independently of antioxidant capacity (42). According to the review published by Johnston and Korolenko (50), the polysaccharides lower triglyceride levels through the ATGL-(PPAR-)/(PGC-1), 24 (SREBP-1c)-ACC/FAS, and ACC-CPT1 signaling pathways, and promote cholesterol reduction. Unfortunately, although both doses of DE showed a hypolipidemic impact, it is worth mentioning that a low dose of DE (500 mg/kg) had a stronger hypolipidemic impact than a high dose of DE, making it a point for further study and verification in the future.

CVD risk indices, such as CAI and CI, are the best predictors of ischemic heart disease risk (33, 34). The AI reflects the extent of atherosclerotic lesions, while ACE is a recognized biomarker of hypertension (40). The significant elevation in cardiovascular risk factors (CAI, CI, AI, and ACE) observed in the TrX-100 model group aligns with previous findings (30). These findings are due to hyperlipidemia achieved by TrX-100 observed in the present study, leading to atherosclerosis and coronary heart disease. Hyperlipidemia and lipid abnormalities are regarded as significant contributors to atherosclerotic cardiovascular diseases and strokes (51). High levels of TC, TG, and LDL-C are primarily responsible for the onset of coronary heart disease (52). The current work established that AI increased in model rats injected with TrX-100, as reported in a previous study (45). Reactive oxygen species induced by Triton are responsible for lipid peroxidation, a key factor for atherosclerosis (53). Atorvastatin supplementation showed insignificant improvement of CAI, CI, and AI; meanwhile, ACE data were close to those of the negative control, which agreed with the suggestion that the standard drug, statin, significantly decreased ACE activity (54). Oral treatment with atorvastatin effectively manages hyperlipidemia and significantly reduces AI, subsequently reducing the risk factor for cardiovascular diseases (45, 49). Atorvastatin might have reduced ACE activity by inhibiting HMG-CoA reductase (39).

The significant reduction in predisposing CVD risk (CAI, CL, AI, and ACE) achieved through 1,000 mg/kg DM supplementation was consistent with (54), who reported that lipid profile and ACE activity, which are crucial for blood pressure regulation, were considerably reduced by the flavonoid-rich fraction of *Hyphaene thebaica*. The present data demonstrate that *H. thebaica* has ACE inhibitory and anti-hyperlipidemic properties, as supported by the work (40). This finding may be attributed to the potassium content of *H. thebaica*, which helps maintain stable blood ACE levels (55). The reduction in the risk factor of CVD associated with a high dose of DM is owing to the antioxidant properties of flavonoids and the phenolic components of DM, as recorded in the present study. These properties act by inhibiting endothelial dysfunction and maintaining the redox balance of normal body cells (56). The mild reduction in all indices predicted CVD and ACE, as demonstrated in the 500 mg/kg DM group, indicated that it is dose-dependent, and the CVD risk is correlated to the lipid profile data obtained in the present study. Since a high dose of DM had more potent hypolipidemic properties than a low dose, which contributed to the improvement of CHD, the suggestion is confirmed by many previous studies (57). Elevating plasma lipids increases CVD risk (16). The prevalence of hypertension is associated with dyslipidemia (1, 3). Elevated levels of LDL-C can trigger the development of atherosclerotic plaques (55). Oxidative stress induced by elevated blood lipid levels leads to endothelial dysfunction and subsequent oxidative modification of LDL-C. This oxidized LDLC accumulates and activates scavenger receptors on macrophages, leading to macrophage activation and the uptake of oxidized LDL-C (1, 57).

The rats receiving 500 mg/kg DE reduced the CVD risk significantly by rebalancing CAI, CI, AL, and ACE. Nevertheless, the flavonoids and phenolic compounds determined in the present study were found to have low levels; the benefits achieved with DE may be associated with the mannan content of DE, as mannose supplementation has been shown to prevent the development of atherosclerosis by controlling the composition of the gut microbiota and suppressing pro-inflammatory monocytes/macrophages (42). D-mannose prevents the adverse effects of a high-cholesterol diet by altering gut microbiota and exhibiting anti-inflammatory properties (58). Interestingly, a high dose of DE revealed a mild improvement in CVD risk despite its mannan content. The finding may be due to the mannose elevation in the blood that could be accompanied by a progressive risk of coronary disease, heart failure, and mortality (41). The observation creates a new channel for future research to determine the blood and *Hyphaene thebaica* mannose content to confirm DE safety and reliability.

The impairment of kidney function, as shown in a group injected with TrX-100, was manifested by increases in creatinine, urea, and uric acid levels significantly compared to the standard control, as confirmed in a previous study (22) that used TrX-100 and (59) that used Triton WR-1339. The elevation of blood creatinine, urea, and uric acid recorded in the TrX-100 model group indicated the development of renal dysfunction (60), owing to TrX-100 oxidative stress, one of the main physio-pathological processes of hyperlipidemia-induced kidney damage (61). The main risk factor for CKD is hyperlipidemia, which implies that poor renal function is caused by lipid buildup in the renal parenchyma (62) and damages glomerular podocytes (63). High TG, TC, and LDL-C levels were associated with kidney disease (64). Atorvastatin administration normalized blood creatinine, urea, and uric acid levels, which may be attributed to the improvement of lipid profiles observed in the same group (1).

The treatment group that received a high dose of DM (1,000 mg/kg) exhibited the best renal performance among all treatments. This was evidenced by the normalization of blood creatinine, urea, and uric acid levels, which resembled those observed in the atorvastatin-treated group, a standard drug. The finding confirmed the nephroprotection % calculated in the present study, which revealed that a high dose of DM (1,000 mg/kg) had the best nephroprotective effect compared to other treatment groups, as previously reported (65). Feeding rats DM powder for 8 weeks significantly improved kidney function, as indicated by reductions in urea, creatinine, and uric acid levels (48). In contrast, the low dose of DM (500 mg/kg) resulted only in a significant decrease in creatinine, indicating that this dosage was insufficient to protect against the development of kidney dysfunction and further highlighting its dose-dependent nature. The improvement in kidney function markers is attributed to the administration of a high dose of DM, owing to its bioactive and antioxidant constituents (13). Unfortunately, DE at two doses had a minor impact on kidney function, normalized creatinine levels, while causing a nonsignificant downgrade in blood urea and uric acid levels.

A significant reduction in kidney tissue antioxidants, GSH-Px, SOD, and CAT, accompanied by elevated MDA levels, was observed in the hyperlipidemic group injected with TrX-100, consistent with previous findings (22). There was a significant increase in oxidative stress during TrX-100 administration, evidenced by depletion of SOD and CAT, and an increase in MDA formation (45). These findings may be attributed to hyperlipidemia induced by TrX-100 exposure, which created oxidative stress, increased ROS formation, and decreased the activities of antioxidant enzymes (59).

The positive effect of atorvastatin administration was reflected in the restoration of antioxidant enzyme activities (GSH-Px, SOD, and CAT) and a reduction in lipid peroxidation (MDA), findings that align with those reported previously (54). Hyperlipidemia, as observed in the TrX-100 model group, as reported in the current work, leads to oxidative stress. Atorvastatin lowers cholesterol levels by inhibiting HMG-CoA reductase (39) and reduces oxidative stress by enhancing GSH-Px, SOD, and CAT activities while decreasing MDA levels.

The aqueous extract of 1,000 mg/kg DM significantly rebalanced GPx, SOD, and CAT activities, as well as MDA. Meanwhile, 500 mg/kg of DM revealed a moderate improvement in antioxidant activity, evidenced by a significant elevation in GPx and an inhibition of MDA, along with a nonsignificant increase in SOD and CAT. This finding may be due to the enhanced antioxidant properties associated with increased doum prehension (66). Many researchers have confirmed the benefits of high-dose DM supplementation (54). The antioxidant system (GSH-Px, GST, and CAT) was corrected by pretreatment of the *H. thebaica* extract (13). *Hyphaene thebaica* is a source of powerful antioxidants, antimicrobials, antidiabetics, antihypertensives, and hypolipidemics (40, 66). Despite the administration of 500 mg/kg DE achieving a marked improvement in antioxidant enzyme activities and a decrease in MDA, 1000 mg/kg DE recorded non-significant improvements in these parameters, which confirm the results of the lipid profile obtained in the present study.

Generally, the benefits achieved with DM 1000 mg/kg, due to the presence of phenolic and flavonoids identified in the present study, which act as antioxidants to control hyperlipidemia, are in agreement with recent work (29, 40). The phytochemical constituents of DM detected by HPLC in the current work revealed its bioactive components, including catechol, catechin, P-hydroxybenzoic acid, vanillic acid, rutin, ferulic acid, and syringic acid. These substances possess several different bioactive characteristics, including anti-inflammatory, antioxidant, and hyperlipidemia effects (15, 67). These biological activities may help restore animals’ serum lipid profiles to normal and protect against risk factors for renal dysfunction and cardiovascular disease (CVD).

Regarding the histopathological observation, unfortunately, to our knowledge, no published paper has studied the histological examination of renal tissue in supplemented *Hyphaene thebaica*. Nevertheless, the kidney specimens examined aligned with the results obtained for nephroprotection and kidney antioxidant status recorded in the current study. In the TrX-100-injected model group, kidney impairment was evidenced by elevated serum levels of creatinine, urea, and uric acid; reduced renal antioxidant activities; and histopathological changes, including congestion of renal blood vessels, degeneration of vacuolated epithelial cells, and perivascular inflammatory cell infiltration. Meanwhile, DM at a high dose of 1,000 mg/kg achieved the best renal performance by normalizing blood creatinine, urea, and uric acid levels, provided the highest nephroprotection%, and rebalanced kidney antioxidant activities. It also showed a typical histological architecture of renal specimens, indicating benefits on kidney function. On the other hand, administration of DE at low and high doses resulted in comparable effects on kidney histology, as evidenced by degeneration of the vacuolar epithelial lining in some renal tubules. This histological finding is consistent with the similar outcomes observed for kidney function parameters and total nephroprotection % in both DE doses.

Notably, administration of DE at both tested doses induced discernible renal histological alterations, evidenced by degeneration of the vacuolar epithelial lining in select renal tubules. This histological finding aligns with the comparable effects observed in biochemical kidney function parameters and the calculated nephroprotection percentage, which indicate renal dysfunction.

Importantly, despite these renal histological changes, DE demonstrated a clear hypolipidemic effect, as reflected by improved lipid profiles and enhanced antioxidant biomarkers. This contradiction between biochemical benefits and local histological structural protection supports the hypothesis that DE’s hypolipidemic and antioxidant effects, likely mediated by its mannan content, do not directly translate into complete histological preservation against TrX-100-induced tubular injury. Consequently, while DE shows promise as a hypolipidemic agent, further investigation is required to elucidate its precise mechanisms, safety profile, and therapeutic potential.

## 5 Conclusion

Administration of DM at a high dose (1,000 mg/kg) achieved an excellent hypolipidemia effect and has mitigated surrogate markers of cardiovascular problems and renal dysfunction risk, including rebalancing CAI, CI, AI, and ACE, normalizing creatinine, urea, and uric acid with the highest nephroprotection%, and mitigating the oxidative stress on renal tissue. Conversely, a low dose of DM (500 mg/kg) had only a mild effect, indicating that DM had a dose-dependent response. Interestingly, a low dose of DE had a more pronounced effect than a high dose. The low dose of DE provided a better hypolipidemic impact, significantly lowering CVD risk by rebalancing CAI, CI, AL, and ACE, and improving antioxidant activities, than a high dose, making it a point for future research on DE safety and reliability, taking into account the mannose content, which is the limitation of the current study. Further studies are needed to confirm whether these biochemical improvements translate into clinical cardiovascular protection.

## 6 Research limitations

The authors recognized that mannose analysis of *Hyphaene thebaica* should have been performed and considered this a research limitation. In discussing some of the existing results, particularly those related to doum endosperm, the authors relied on the mannose content mentioned in recently published research to justify parts of the findings. Accordingly, in future research, the authors advise analyzing the mannose content.

## Data Availability

The raw data supporting the conclusions of this article will be made available by the authors, without undue reservation.
